# Species-selective activation of a β-galactosidase fluorogenic probe

**DOI:** 10.1038/s41598-025-08443-y

**Published:** 2025-07-02

**Authors:** Kaja Jaskot, Angelika Mielcarek, Łukasz Popenda, Ahmet Kertmen, Bartosz F. Grześkowiak, Jan Barciszewski, Patrick M. Perrigue

**Affiliations:** 1 NanoBioMedical Centre, Adam Mickiewicz University in Poznań, Wszechnicy Piastowskiej 3, Poznań, 61-614 Poland; 2 Faculty of Physics and Astronomy, Adam Mickiewicz University in Poznań, Uniwersytetu Poznańskiego 2, Poznań, 61-614 Poland; 3 Institute of Bioorganic Chemistry, Polish Academy of Sciences, Noskowskiego 12/14, Poznań, 61-704 Poland

**Keywords:** Molecular modelling, Hydrolases, Senescence, Fluorescent dyes, Biochemistry

## Abstract

Fluorogenic probes capable of distinguishing enzyme activity between species are crucial for precise detection in complex biological environments. Herein, we present 5-(Pentafluorobenzoylamino) Fluorescein di-β-D-galactopyranoside (PFB-FDG), a substrate selectively activated by human β-galactosidase (β-gal) without cross-reactivity with bacterial β-gal. We tested PFB-FDG under various control conditions using purified enzymes and live cells to demonstrate that it does not activate in the presence of bacterial β-gal. Moreover, molecular docking studies suggest that the selectivity results from steric hindrance within the smaller binding pocket of bacterial β-gal, in contrast to the larger binding pocket of the human enzyme. This species-selective probe represents a major advancement, with its unique properties providing a valuable tool for future studies on human-bacterial interactions.

**Supplementary Information:**

The online version contains supplementary material available at 10.1038/s41598-025-08443-y.

## Introduction

β-galactosidase (β-gal) is an enzyme classified as a glycoside hydrolase (GH) and is evolutionarily conserved across diverse organisms^1,2^. Its main function is to hydrolyze the terminal non-reducing β-D-galactose units in β-D-galactosides. To monitor β-gal in cells or organisms, many different types of synthetic substrates have been developed that can assess its activity^3^. The basic design of these substrates contain galactose, which the enzyme recognizes, conjugated to a chemical moiety that produces color upon cleavage. Notable examples of chromogenic substrates include ONPG (ortho-Nitrophenyl-β-galactoside)^4^ and X-gal (5-Bromo-4-chloro-3-indolyl-β-galactopyranoside)^5^. Additionally, these substrates include the β-glycosidic linkage, a key feature in the structure of lactose, which is essential for β-gal to perform its function.

Furthermore, various synthetic substrates have been designed to incorporate fluorescence, enhancing their utility in enzymatic studies. Fluorescein di-β-D-galactopyranoside (FDG) is a synthetic fluorogenic probe for detecting β-gal activity^6,7^. FDG is non-fluorescent, consisting of fluorescein conjugated to two galactose groups. β-gal cleaves the glycosidic bonds, releasing free fluorescein, which can be detected as green fluorescence when excited with a 488 nm laser. FDG is highly versatile, enabling the detection of β-gal activity across different species^8^. The bacterial version of β-gal, encoded by the lacZ gene, is commonly used as a reporter gene, with FDG utilized to track gene expression in real-time^9,10^. Detecting β-gal activity is also important for diagnosing cancers^11^. Notably, one of the distinct changes associated with cellular senescence is the significant elevation of SA-β-gal (senescence-associated β-galactosidase) activity^12,13^. This increase comes from lysosomal β-gal encoded by Galactosidase, beta 1 (GLB1). As a key biomarker, SA-β-gal activity is crucial for exploring mechanisms of aging and is detectable using FDG.

To improve substrate delivery and enhance detection in live cells, different versions of β-gal probes based on FDG have been developed. 5-(Dodecanoylamino) Fluorescein di-β-D-galactopyranoside (C12-FDG), features a lipid tail that improves its ability to penetrate live cells, enabling more sensitive measurement of β-galactosidase activity in live-cell assays^8^. SPiDER-βGal is another cell-permeable fluorescent probe when hydrolyzed by β-gal generates a reactive quinone methide intermediate that covalently attaches to nearby intracellular proteins^14^. This self-immobilization process ensures that the fluorescent signal remains within the cell. Other advancements include the design of β-gal-activatable probes with improvements in their spectral characteristics. For example, 4-Methylumbelliferyl-β-D-galactopyranoside emits blue fluorescence at approximately 450 nm^15^while Resorufin β-D-galactopyranoside emits red fluorescence at around 580 nm^16^. Recent advancements in near-infrared probes further expand the range of available tools for tracking β-gal activity in vivo^17,18^. These innovations build upon various strategies aimed at improving probe functionality.

PFB-FDG is another modified derivative of FDG optimized for enhanced cell uptake and retention. The PFB modification forms a covalent bond with thiol groups of intracellular proteins through aromatic nucleophilic substitution involving at least one fluorine atom. This effectively immobilizes it within the cell. PFB-FDG has been utilized in the study of cellular aging^19^. FDG and some of the other described variants of FDG are thought to all function similarly in terms of their activation across different organisms. However, PFB-FDG stands out in that, to our knowledge, it has not been reported in any bacterial system. We hypothesized that the relatively bulky structure of PFB-FDG may impede efficient access to the active site of β-gal derived from bacteria. While the PFB modification addresses improving the properties of the FDG substrate, its species-selectivity remains uncharacterized. Our study aimed to establish PFB-FDG as a species-selective probe. In environments where human cells and bacteria coexist and interact, distinguishing between β-gal activities originating from different sources becomes challenging as conventional substrates react with both. Species-selective probes for SA-β-gal offer significant advantages for studying cellular senescence in the presence of bacteria^20^. Hence, PFB-FDG specifically targeting human β-gal can minimize cross-reactivity and enhance accuracy.

## Results

**Fig. 1:**
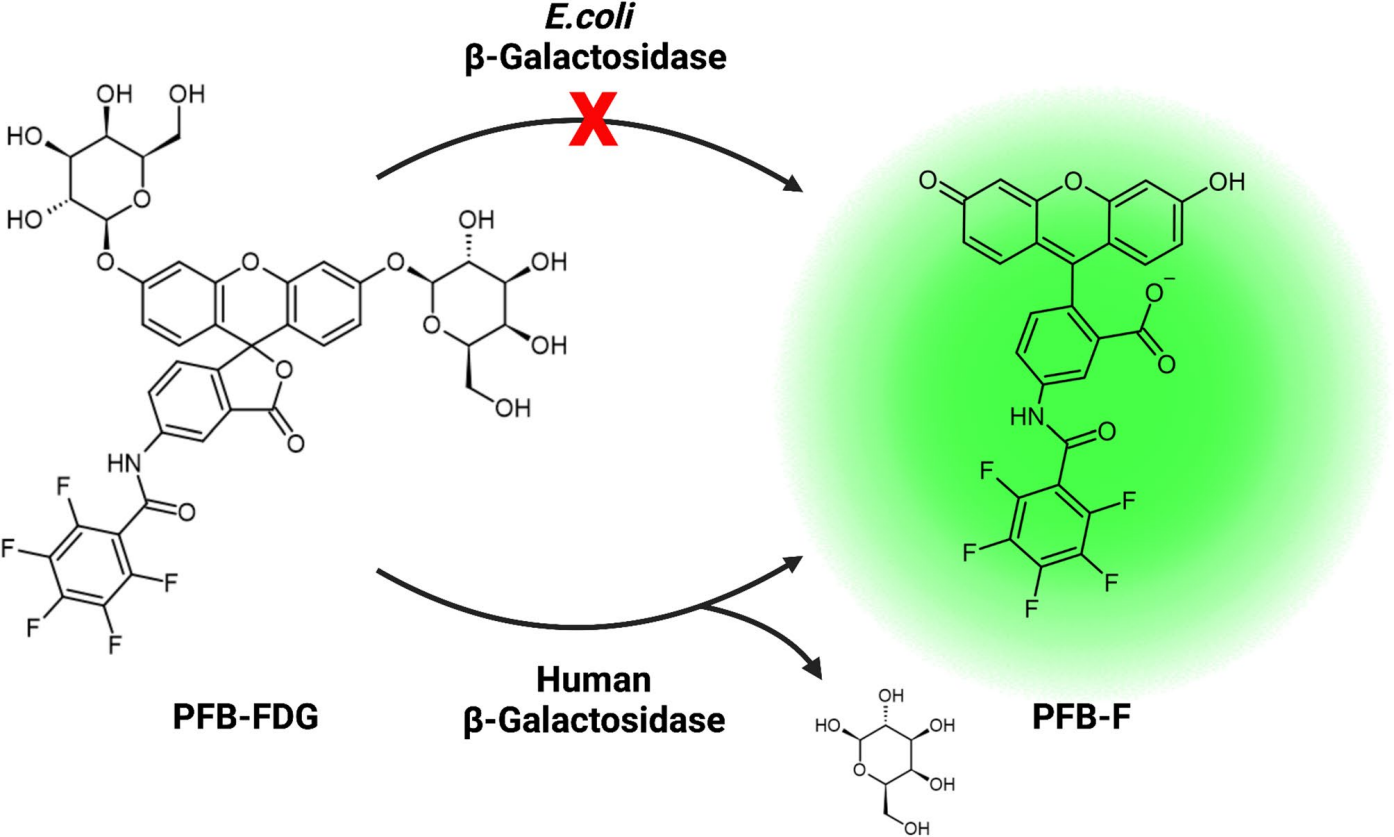
Illustration of the enzymatic cleavage of PFB-FDG into PFB-fluorescein (PFB-F), highlighting the difference in substrate activation.

**Fig. 2:**
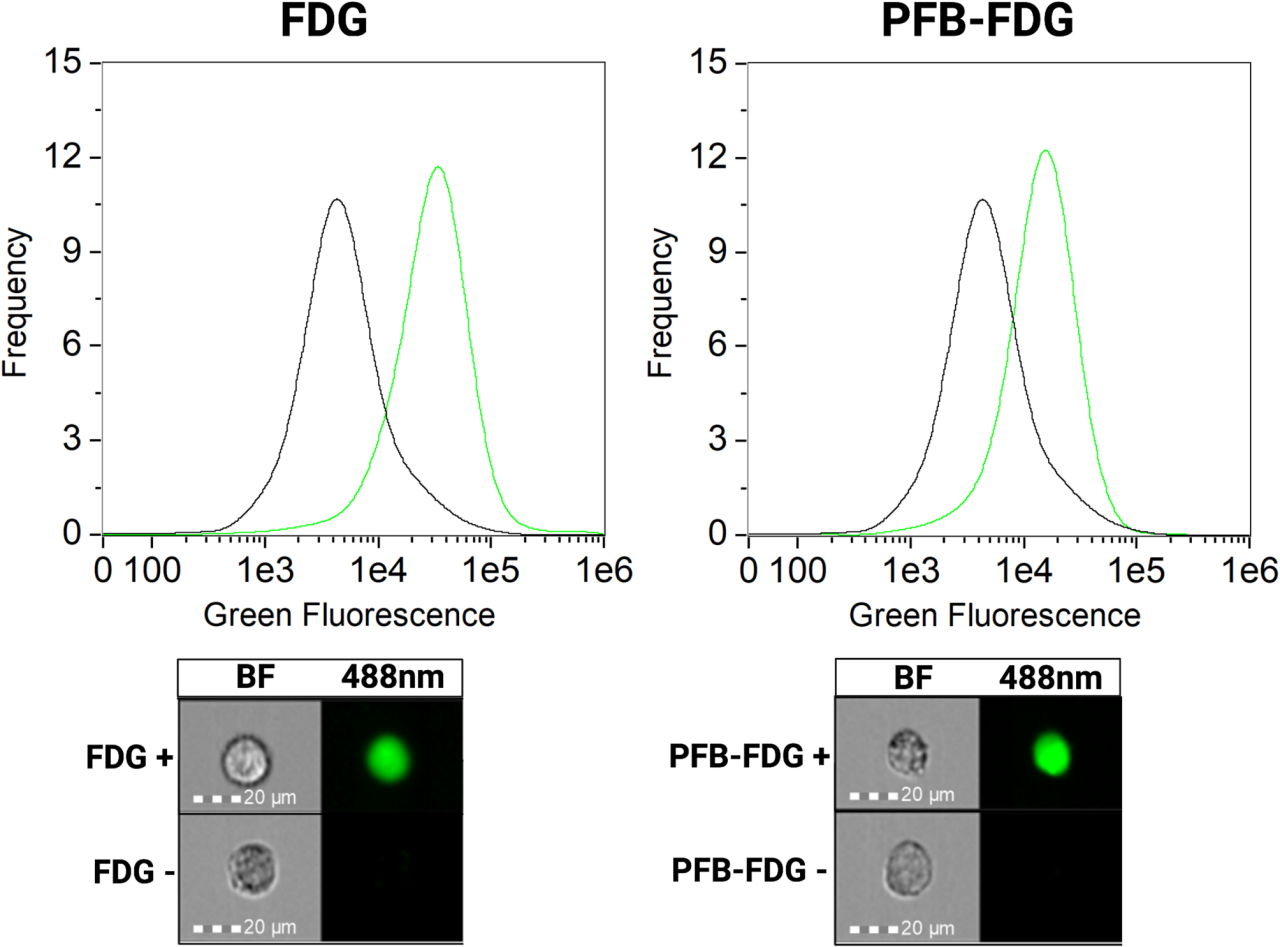
Comparison of FDG and PFB-FDG activation in human U251.JMJD3wt cells expressing SA-β-gal, analyzed using imaging flow cytometry. Histograms show green fluorescence intensity for samples with (green) and without (black) the probe. Representative images of the cells are displayed below each histogram for BF = Brightfield channel, and 488 nm = green channel.

**Fig. 3:**
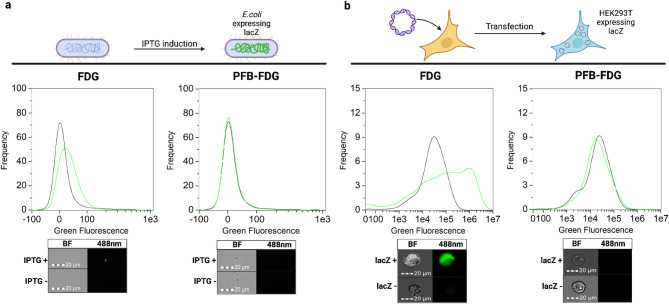
(**a**) Comparison of probe activation in *E. coli* cells with (green) or without (black) induced lacZ expression by IPTG. (**b**) Comparison of probe activation in HEK293T cells transfected with (green) or without (black) the lacZ plasmid. Representative images of the cells are displayed below each histogram for BF = Brightfield channel, and 488 nm = green channel. Graphics made using BioRender.com.

Here, we demonstrate that PFB-FDG exhibits species-selectivity for human β-gal over the bacterial β-gal from *E. coli* (Fig. 1). Our investigation began with examining the activation of FDG and PFB-FDG in human cells expressing endogenous SA-β-gal activity. We observed green fluorescence, demonstrating both probes are activated by human β-gal (Fig. 2). Following this, we conducted tests on *E. coli* cells to assess probe selectivity. To induce the expression of β-gal in *E. coli* we used isopropyl β-D-1-thiogalactopyranoside (IPTG)^21^. The upregulation of β-gal expression was confirmed using FDG, however, no activation was observed when PFB-FDG was incubated with the same, β-gal-expressing bacteria (Fig. 3a). To further demonstrate the inability of *E. coli* β-gal to activate PFB-FDG, we used human cells that were transfected with a plasmid encoding the lacZ gene. The expression of lacZ in these cells was confirmed with FDG. In contrast, PFB-FDG showed no activation under those tested conditions (Fig. 3b).

To rule out the possibility that the bulky PFB group may impede PFB-FDG from entering live bacterial cells, we prepared cell lysates and incubated them with the different probes. While FDG was activated by *E. coli* lysate, PFB-FDG remained inactivated (Fig. S1). In contrast, human cell lysates containing SA-β-gal activated PFB-FDG consistent with our findings in live cells. We also tested purified β-gal from different sources (Fig. S2). Purified *E. coli* β-gal successfully activated FDG but failed to activate PFB-FDG. In contrast, β-gal isolated from bovine liver, which shares highly conserved sequence homology with the human enzyme, was able to activate PFB-FDG. These data demonstrate that PFB-FDG is selective and not activated by *E. coli* β-gal.

We also screened additional β-gal sources from various organisms for their ability to activate FDG, PFB-FDG and 5-(Acetamido) Fluorescein-di-β-D-galactopyranoside (AA-FDG), a structurally related probe to PFB-FDG that lacks the bulky aromatic ring. The tested enzymes included β-gal from *Kluyveromyces lactis* (yeast), *Aspergillus oryzae* (filamentous fungus), and a lysate from a probiotic supplement containing lactose-metabolizing bacteria commonly found in the human gut: *Lactobacillus acidophilus*,* Lactobacillus rhamnosus*,* Bifidobacterium lactis*, and *Lactobacillus plantarum* (Fig. S3). β-gal from *E. coli*,* K. lactis*, and the probiotic supplement lysate activated FDG and AA-FDG, but failed to activate PFB-FDG. The activation of AA-FDG by these β-gal sources adds more weight to the findings that steric hindrance plays a key role in preventing PFB-FDG activation. β-gal from *A. oryzae* and bovine β-gal activated all the probes. This suggests that *A. oryzae*, bovine, and human β-gal possess structural features that accommodate bulkier substrates.

**Fig. 4:**
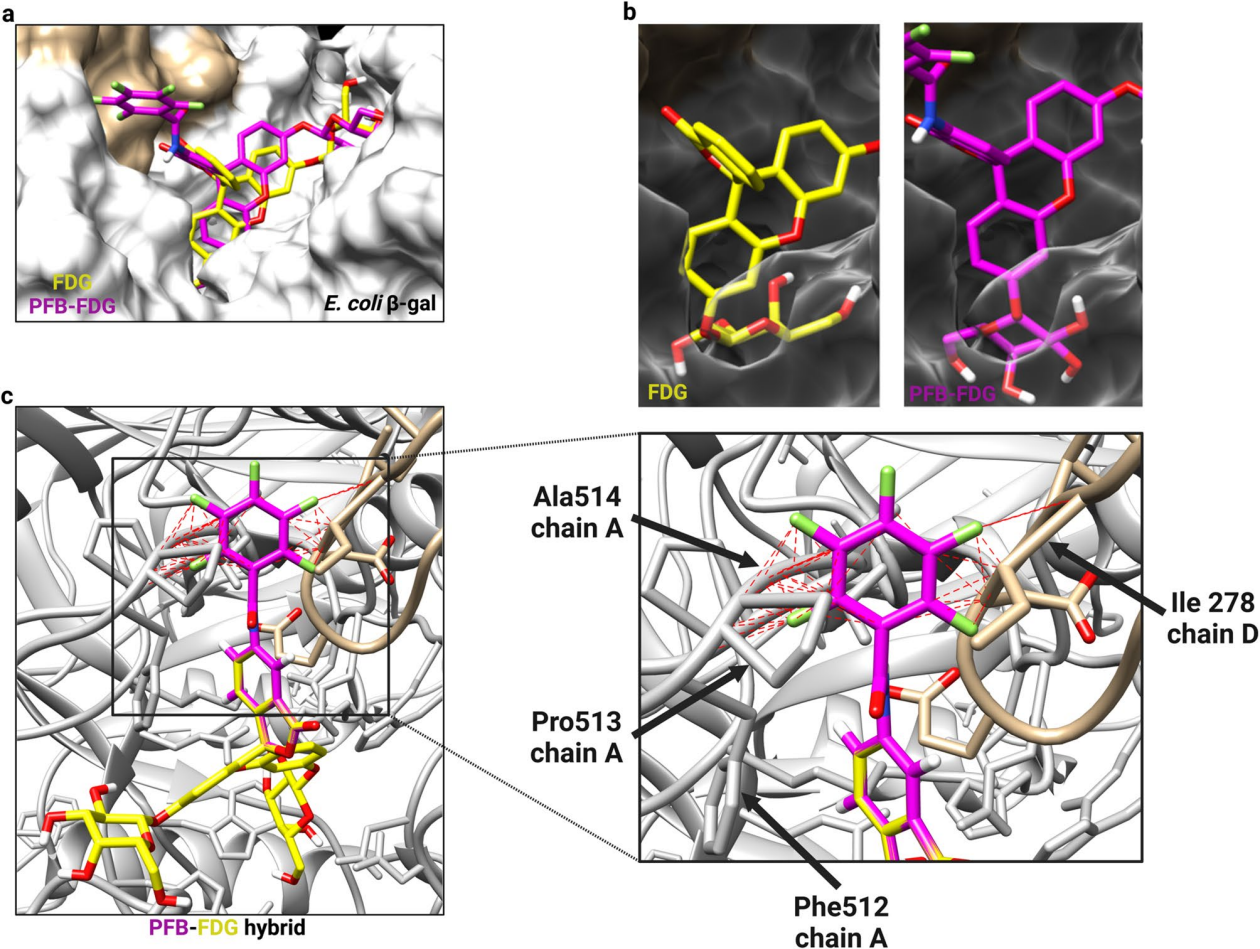
Molecular docking analysis of FDG and PFB-FDG with *E. coli* β-gal. (**a**) The superimposed docking positions of FDG (−9.386 kcal/mol) and PFB-FDG (−11.225 kcal/mol) within the binding pocket. (**b**) View of the active site with the protein surface made transparent to show the different orientations of the probes. (**c**) *Left Panel*: The docking pose of FDG in shallow mode is shown, with a grafted PFB group forming a PFB-FDG hybrid structure. Red dashed lines indicate steric clashes between the PFB group and nearby amino acid residues in chains A and D. These clashes highlight the structural incompatibility introduced by the PFB group in this binding configuration. *Right Panel*: A zoomed-in view of the clash points from the left panel, with labels indicating the specific amino acids and their positions.

To investigate the basis of species selectivity for PFB-FDG, we examined the structural properties of *E. coli* β-gal, which functions as a tetramer with each monomer possessing hydrolytic activity^22,23^. We performed molecular docking of FDG and PFB-FDG onto *E. coli* β-gal, specifically targeting one of the substrate binding pockets located at the interface between monomer chains A and D (Fig. S4 and S5). The docking results for both probes when superimposed shows FDG is more embedded within the binding pocket with the glycosidic bond of PFB-FDG offset by 1.654 Å from that in FDG (Fig. 4a and b).

*E. coli* β-gal undergoes a two-step sequence for substrate binding, initially involving recognition and alignment with specific amino acid residues, followed by stabilization within the active site through additional residue interactions^24^. When a substrate initially enters the binding pocket, it interacts with the upper region of the active site, referred to as the shallow mode, where Trp999 plays a crucial role in the initial binding^25^. Subsequently, the substrate moves deeper into the pocket for the reaction, known as the deep mode. In deep mode, Glu461 and Glu537 are key residues for catalysis^26–28^. We visualized the two-step binding of FDG using 2D ligand-protein interaction diagrams. The first-ranked model for FDG (−9.919 kcal/mol), identified based on the lowest binding free energy, positions FDG in shallow mode, establishing critical contacts with the aromatic side chain of residue Trp999 (Fig. S6a). The second-ranked model (−9.386 kcal/mol) is in deep mode and shows that FDG forms hydrogen bonds with the catalytic residues, with the distance from atom O15 to Glu431 measuring 2.92 Å, and the distance to Glu537 measuring 2.95 Å (Fig. S6b). In contrast, PFB-FDG exhibits significant differences in contact points primarily due to the inverted positioning of its sugar inside the binding pocket (Fig. S6c). This is most evident when comparing the oxygen atom at position O2 in PFB-FDG, which corresponds to atom O15 in FDG. Altogether, these data suggest that PFB-FDG is not positioned within the binding pocket in the same orientation as FDG.

We also performed further analysis focusing on key amino acid residues to track the movement of substrate from shallow to deep modes. FDG progresses from the shallow to deep mode with a continuous and well-aligned binding trajectory. (Fig. S7 and Supplementary Videos 1 and 2). PFB-FDG does not achieve a deep mode like FDG due to the misalignment of contacts made with Trp999, Glu461, and Glu537. To investigate this further, we docked FDG in the shallow mode and then grafted a PFB group onto it to identify potential clash points (Fig. 4c). The grafted PFB group encounters several clashes with residues Phe512, Pro513, and Ala514 in chain A, as well as Ile278 in chain D, suggesting that there is steric hindrance obstruction to the optimal positioning in shallow mode. Altogether, this data suggests that the bulky PFB group interferes with the stepwise movement of the substrate for correct binding inside the pocket. The binding pocket of human β-gal has been already described^29^with structural comparisons revealing significant variations in its amino acid sequences compared to *E. coli* β-gal. Li et al. calculated an RSMB value of 6.236 Å between the domains of *E. coli* and human β-gal^20^. Notably, the binding pocket volume of *E. coli* β-gal (457.2 Å^3^) is smaller than that of human β-gal (280.1 Å^3^)^20^. Glu461 and Glu537 in *E. coli* β-gal correspond to Glu188 and Glu268 in human β-gal, and these key residues contribute to the enzymes having the same catalytic mechanism. However, sequence variation in the extensible region and differences in binding pocket volume contributes to the species-selectiveness of PFB-FDG. We performed the same molecular docking procedures for human β-gal as we did for *E. coli* β-gal using both probes (Fig. S8-S10). The results indicate that both FDG and PFB-FDG adopt similar binding conformations within the binding site, which is consistent with their activation by human β-gal. Altogether, these data suggest that PFB-FDG activation is dependent on specific structural features of the β-gal enzyme that differ between species.

## Discussion

Enzymes can exhibit multi-level specificity toward their substrates, enabling precise biochemical recognition. At the most fundamental level, glycosidases demonstrate anomeric specificity. Those that hydrolyze β-glycosidic bonds, such as β-gal, are highly specific and do not act on α-linked substrates^30^. A further layer of specificity involves recognition of the monosaccharide type. β-gal selectively recognizes galactosyl residues and does not act on glucosyl substrates, which functionally separates it from β-glucosidase^31^. These classical substrate-specific mechanisms are well established and form the basis for designing fluorogenic probes such as FDG. Our study reveals an additional and underexplored level of specificity — species selectivity.

To explain the selective hydrolysis of PFB-FDG between species, it is important to consider the structural and evolutionary differences among the β-gal enzymes tested. Although *K. lactis* and *A. oryzae* are both fungi, their β-gal enzymes differ substantially in GH family classification and structure, which likely accounts for their distinct reactivity with bulky probes like PFB-FDG. β-gal enzymes are classified into GH families 1, 2, 35, and 42 based on sequence and function^32^. The β-gal enzymes from *A. oryzae* and mammals belong to GH35, whereas *K. lactis* is an exception among fungi with its β-gal enzyme belonging to GH2, a family mostly composed of bacterial enzymes, including that of *E. coli*. Pereira-Rodríguez et al. solved the X-ray crystal structure of *K. lactis* β-gal, revealing that, while it shares the typical GH2 fold seen in bacterial enzymes, it possesses a notably narrow channel to its active site^33^. This narrow channel likely prevents PFB-FDG from accessing the active site and consistent with our data. In contrast, the *A. oryzae* β-gal shares high structural similarity with human β-gal (RMSD = 1.002 Å)^20^. Together, these structural differences provide an explanation for why PFB-FDG is reactive with *A. oryzae* β-gal, but not with *K. lactis* β-gal, despite their common fungal origin. While our results strongly support species-selective activation of PFB-FDG by human β-gal, it appears to extend broadly to other mammalian and potentially further to other fungal sources of β-gal. Our data also demonstrate a consistent lack of PFB-FDG activation by the bacterial β-gal enzymes we tested, suggesting that this lack of reactivity may be a more universal among bacterial species. To better define the scope and limitations of PFB-FDG selectivity, future studies should explore a broader range of β-gal enzymes across GH families, with a focus on enzymes representing structural intermediates or evolutionary outliers.

We investigated the impact of substituents at the 5-position of FDG probes. All the β-gal enzymes included in our study hydrolyzed FDG and AA-FDG. This data suggests that selective hydrolysis is introduced by bulky 5-position substituents, rather than by their presence alone. Other studies using FDG-based probes bearing 5-position substituents corroborate our study. For example, chloromethyl-FDG (CMFDG) is a modified form of FDG that includes a reactive chlorine atom. This probe has been shown to be activated in lacZ-expressing cells^34^. Interestingly, C12-FDG, which contains a lipophilic tail substituent at the 5-position, is hydrolyzed by *E. coli* β-gal^35,36^. This suggests that the long carbon chain is flexible enough to adopt conformations that avoid steric hindrance. These diverse examples of other 5-position substituents activated by *E. coli* β-gal further reinforce the observed structure–activity relationship observed for PFB-FDG.

The research development of specialized substrates tailored for β-gal is ongoing. Some of these advanced chemical probes are engineered to be species-selective, recognizing and binding to the β-gal expressed in human cells with high fidelity. As a result, they avoid cross-reactivity with bacterial β-gal expressed from the lacZ gene. Species-selective probes for SA-β-gal could potentially allow for precise studies of microbial dynamics and their impact on host health during aging^37^. In the context of anti-aging therapies that focus on eliminating senescent cells, PFB-FDG may help to facilitate the evaluation of new drugs^38^. Furthermore, our study shows that steric hindrance contributes to the observed selectivity of PFB-FDG. These findings may provide a basis for the molecular design of new probes and β-gal-responsive prodrugs aimed at targeting senescent cells^39^. In the future, such therapeutics could be engineered to minimize off-target effects, reducing the risk of premature activation by commensal microbiota. Our study demonstrates that PFB-FDG is a species-selective probe, challenging the previous assumption that it can be activated by β-gal from all species. This discovery underscores the need for further research into its applications, particularly in contexts requiring precise species-selective activation.

## Methods

### Preparation of chemical stocks

FDG (F2756, Sigma, St. Louis, MO, USA), AA-FDG (AL854, Synthose, Concord, ON, Canada) and PFB-FDG (7230, Setareh Biotech, LLC, Eugene, OR, USA) were each prepared as 10 mg/mL stock solutions in DMSO (D2438, Sigma, St. Louis, MO, USA) and stored at −20 °C.

### Bacteria and human cell culture

The U251 human glioma cell line and HEK293T human epithelial-like kidney cell line were obtained from the American Type Culture Collection (ATCC, Manassas, VA, USA). The U251.JMJD3wt cell line was generated from U251 cells and engineered to overexpress the histone demethylase JMJD3, as previously described^40^. Cells were cultured in Dulbecco’s Modified Eagle’s Medium (DMEM; D0822, Sigma, St. Louis, MO, USA) supplemented with 10% fetal bovine serum (FBS; F7524, Sigma, St. Louis, MO, USA), and antibiotic/antimycotic solution (A5955, Sigma, St. Louis, MO, USA). The culture medium was filtered through a 0.22-micron PES disc syringe filter (S2GPU05RE, Millipore, Burlington, MA, USA). All cell cultures were maintained in a humidified incubator at 37 °C with 5% CO_2_. The *E. coli* strain was sourced from ATCC (35218, Manassas, VA, USA). *E. coli* was cultured in 5 mL of LB medium (Lennox; BioShop Canada Inc., ON, Canada) supplemented with 0.1% ampicillin. 100 µM of IPTG (BioShop Canada Inc., Cat. No. 367-93-1, ON, Canada) was added to LB medium to induce lacZ expression in bacteria, with a 4 h incubation.

### Imaging flow cytometry

Live *E. coli* in a 5 mL culture tube and human cells in a 6-well format were incubated with a final concentration of 3 mg/mL for 2 h. For the *E. coli* cultures, cells were harvested by centrifugation at 1200 rpm for 5 min. The resulting cell pellet was resuspended in 200 µL of PBS and then further diluted 1:100 in PBS before analysis by IFC. Human cell cultures were washed three times with HBSS (14175053, Gibco, Waltham, MA, USA) and then incubated with trypsin at 37 °C for 3 min. After trypsinization, cells were centrifuged at 1200 rpm for 5 min and the pellet was resuspended in 100 µL of PBS. All cells were analyzed using an Amnis FlowSight Imaging Flow Cytometer. Histograms and images in the figures were generated using IDEAS software^41^.

### DNA transfection

pSV-β-Galactosidase Control vector (E1081) containing the lacZ gene was obtained from Promega (Madison, WI, USA). HEK293T cells were seeded into 6-well plates in DMEM without FBS. Each well was transfected with 2.5 µg of DNA plasmid using 5.0 µL of lipofectamine 2000 (Thermo Fisher Scientific,8224 Waltham, MA, USA). After 4 h the cell culture medium was replaced with DMEM containing FBS. At 24 h post-transfection, the cells were incubated with probes for 2 h, followed by preparation for IFC analysis.

### Cell lysate Preparation and enzymatic assay

All lysates were prepared using a lysis buffer containing 0.2% SDS (L-4509, Sigma, St. Louis, MO, USA) and protease inhibitor cocktail (11836153001, Roche, Basel, Switzerland). *E. coli* bacteria lysates were prepared from a culture with OD value of 0.27–0.32. 500 µL was centrifuged at 1400 rpm for 5 min and the resulting pellet was resuspended in 100 µL of the lysis buffer. For the preparation of human cell lysates, U251.JMJD3wt cells were trypsinized from a confluent T75 flask and centrifuged at 1400 rpm for 5 min. The supernatant was discarded, and the cell pellet was resuspended in 100 µL of lysis buffer. Probiotic supplement (219104, Dr. Max Pharma s.r.o, Praha, Czechia) was prepared by mixing 100 mg of the capsule contents with 100 µL of lysis buffer.

Enzyme stock solution of β-gal was prepared at 1000 U/mL for E. *coli* (G5635, Sigma-Aldrich, Canada Co., Canada), 2500 U/mL for *A. oryzae* (G5160, Sigma, St. Louis, MO, USA), and 10 U/mL for bovine (G1875, Sigma-Aldrich, Canada Co., Canada). Stock solution of β-gal from *K. lactis* (G3665, Sigma, St. Louis, MO, USA) was purchased as a 50 mL solution with an activity of 2600 U/g.

For the assay, a 96-well plate was prepared by adding 248 µL of PBS, 1 µL of enzyme stock solution and 1 µL of probe stock solution to each well. The reaction was incubated for atleast 2 h at 37 °C. All reaction samples were diluted 1:30 in water and measured using a fluorometer (FluoroSens, Gilden Photonics Ltd, Glasgow, UK), with endpoint fluorescence emission recorded in the 450–600 nm wavelength range following excitation at 488 nm.

### Molecular Docking

Molecular docking simulations were performed using UCSF Chimera^42^ in conjunction with AutoDock Vina^43,44^. The crystal structures of *E. coli* β-gal and human β-gal were obtained from the Protein Data Bank (PDB) with accession codes 1JZ7 and 3THC, respectively. Before docking, the protein structures were prepared by removing amino acids distant from the binding pocket and all heteroatoms and non-native ligands. The structures were then processed using the “Dock Prep” tool in Chimera. The ligand probes were prepared by using the “addH” and “addCharge” options to ensure proper hydrogenation and charge assignment. The search volume was defined to encompass the binding pocket area using AutoDock Vina. After performing the docking, steric clashes and contacts were analyzed using the “Find Clashes/Contacts” tool in Chimera. The figures were created using the solid surface option and transparency settings to improve visual interpretation of the docking results^45^. The grafting of PFB onto the docked FDG was achieved by selecting the atoms between the two and utilizing the “match” option in Chimera. To facilitate proper structural overlap with FDG, a segment of the distal region of fluorescein was used as the grafting point, creating a hybrid docking model. Ligand-protein interactions (Ligplots) were analyzed using LigPlot +^46^. Structures and docked ligands were exported from Chimera as PDB files to PyMOL^47^ prior to being uploaded to LigPlot +.

## Electronic supplementary material

Below is the link to the electronic supplementary material.

Supplementary Material 1

## Data Availability

The data that support the findings of this study are available from the corresponding author, P. M. P., upon reasonable request.
